# Novel Mechanisms of Compromised Lymphatic Endothelial Cell Homeostasis in Obesity: The Role of Leptin in Lymphatic Endothelial Cell Tube Formation and Proliferation

**DOI:** 10.1371/journal.pone.0158408

**Published:** 2016-07-01

**Authors:** Akinori Sato, Ryuta Kamekura, Koji Kawata, Masaya Kawada, Sumito Jitsukawa, Keiji Yamashita, Noriyuki Sato, Tetsuo Himi, Shingo Ichimiya

**Affiliations:** 1 Department of Human Immunology, Research Institute for Frontier Medicine, Sapporo Medical University School of Medicine, Sapporo, Japan; 2 Department of Pathology, Sapporo Medical University School of Medicine, Sapporo, Japan; 3 Division of Breast Surgery, KKR Sapporo Medical Center Tonan Hospital, Sapporo, Japan; 4 Department of Otolaryngology, Sapporo Medical University School of Medicine, Sapporo, Japan; USF Health Morsani College of Medicine, UNITED STATES

## Abstract

Leptin is a hormone produced by adipose tissue that regulates various physiological processes. Recent studies have shown that the level of circulating leptin is elevated in obese patients and have suggested a relationship between obesity and postoperative lymphedema. However, the mechanisms by which postoperative lymphedema develops in obese patients and the mechanisms by which leptin regulates lymphatic endothelial cell homeostasis such as tube formation and cell proliferation remain unknown. Here we report that leptin regulates tube formation and cell proliferation in human dermal lymphatic endothelial cells (HDLECs) by activation of the signal transducer and activator of transcription 3 pathway, which is downstream signaling of the leptin receptor. Additionally, we found that upregulation of suppressor of cytokine signaling 3 underlies the mechanisms by which a high dose of leptin inhibits cell proliferation and tube formation. Leptin also enhanced expression of the proinflammatory cytokine IL-6 in HDLECs. Interestingly, IL-6 rescues the compromised cell proliferation and tube formation caused by treatment with a high dose of leptin in an autocrine or paracrine manner. Taken together, our findings reveal a novel mechanism by which compromised HDLECs maintain their homeostasis during inflammation mediated by leptin and IL-6. Thus, regulating the level of leptin or IL-6 may be a viable strategy to reduce the incidence of postoperative lymphedema.

## Introduction

The lymphatic system is uniquely adapted for continuous removal of interstitial fluid and proteins and also plays an essential role in the immune response by directing antigen-presenting cells from tissues to regional lymph nodes [1]. Although lymphatic endothelial cells have many properties in common with the endothelium of blood vessels, they also have distinct structural characteristics reflecting their specific functions [2–4]. Impairment of lymphatic structure and function results in pathological conditions such as tumor metastasis, autoimmune response alteration and lymphedema [5].

Lymphedema is a condition of localized fluid retention and tissue swelling caused by a compromised lymphatic system. Lymphedema is defined as primary (congenital) or secondary (acquired) chronic tissue swelling. The overall incidence of arm lymphedema in breast cancer patients who underwent axillary lymph node dissection has been reported to be 8% to 56% at 2 years post-surgery [6]. The development of human primary lymphedema is associated with gene mutations such as VEGFR3 and FOXC2 [7–9]. On the other hand, secondary lymphedema is caused by surgery or radiation for cancer treatment, infection or trauma [5]. Additionally, recent studies have suggested a relationship between obesity and development of postoperative lymphedema [10–12]. Physical compression to the lymphatic duct by adipocytes or fibrosis of lymphatic smooth muscles is thought to cause obesity-related lymphedema [13,14]. However, the mechanisms by which postoperative lymphedema develops, especially the role of lymphatic endothelial cells in this process, remain unknown.

Leptin is a hormone produced by adipose tissue that regulates various physiological processes and behaviors including appetite, body weight, neuroendocrine functions and glycemia [15,16]. Increased serum levels of leptin in obese patients have been reported [17,18]. The effects of leptin are mediated via actions on leptin receptors (Ob-Rs) expressed ubiquitously in mammalian tissues [19,20]. Ob-Rs mediate all actions of leptin via activation of multiple intracellular signaling pathways such as Janus kinase (JAK)/signal transducer and activator of transcription (STAT) and mitogen-activated protein kinase (MAPK) [21–23]. There are several lines of evidence indicating that leptin influences vascular endothelial cell homeostasis. Korda et al. reported that leptin increased oxidative/nitroxidative stress in endothelial cells via changes in endothelial NO synthase expression and intracellular L-arginine level [24]. Leptin increases proliferation and reduces apoptosis of human umbilical vein endothelial cells (HUVECs) [25,26]. Additionally, leptin can regulate the immune response by activating immune-competent cells [27]. Previous studies have demonstrated that leptin enhances the expression of proinflammatory cytokines such as IL-6 and IL-8 and regulates inflammatory responses [28,29]. Leptin also induces the production of proinflammatory cytokines such as IFN-γ, but not IL-4, from helper T cells [30]. However, the role of leptin in lymphatic endothelial cell homeostasis has not been clarified.

Here we report an increased incidence of lymphedema in obese patients who received lymph node dissection for treatment of breast cancer. Treatment with a high dose of leptin inhibits HDLEC proliferation and tube formation. In addition, leptin induces phosphorylation of STAT3, which is downstream signaling of Ob-Rs. We also found that upregulation of suppressor of cytokine signaling 3 (SOCS3) underlies the mechanisms by which a high dose of leptin inhibits cell proliferation and tube formation. Interestingly, leptin also enhanced expression of the proinflammatory cytokine IL-6 in HDLECs. IL-6 has the capacity to rescue the compromised cell proliferation and tube formation caused by a high dose of leptin in an autocrine or paracrine manner. These findings reveal novel biological mechanisms by which a large amount of leptin compromises lymphatic endothelial cell homeostasis. Thus, regulating the level of leptin and/or IL-6 may reduce the incidence of postoperative lymphedema.

## Materials and Methods

### Human samples

Sixty-five women with primary breast cancer underwent partial or total mastectomy and axillary lymph node dissection at the KKR Sapporo Medical Center Tonan Hospital during the period from November 2009 to March 2015. After the operation, 12 patients had lymphedema and 53 patients had no lymphedema. Characteristics of the patients are summarized in Table 1. The diagnosis of lymphedema was established on the basis of general symptoms and clinical examination by a doctor. Body mass index (BMI) was calculated using the following formula: BMI = weight (kg) / height (m)^2^.

**Table 1:** Characteristics of the breast cancer patients who underwent partial or total mastectomy and axillary lymph node dissection.

	Objective lymphedema n = 12	No lymphedema n = 53	*p*-value
**Age (years ± SD)**	59.7 ± 14.2	61.9 ± 13.9	n.s.
**BMI ≥ 30 (kg/m**^**2**^**)**	3/12 (25.0%)	4/53 (7.5%)	*p* = 0.035
**25 ≤ BMI < 30 (kg/m**^**2**^**)**	7/12 (58.3%)	9/53 (17.0%)	*p* = 0.024
**ER (>1%)**	6/12 (50.0%)	31/53 (50.2%)	n.s.
**PgR (>1%)**	7/12 (58.3%)	22/53 (41.5%)	n.s.
**LDL-C ≥ 140 (mg/dL)**	5/12 (41.6%)	17/53 (32.1%)	n.s.

BMI, body mass index; ER, estrogen receptor; PgR, progesterone receptor; LDL-C, low-density lipoprotein cholesterol; SD, standard deviation; n.s., not significant.

Levels of estrogen and progesterone in serum were measured by Architect plus i1000SR (Abbott Diagnostics, IL, USA). Low-density lipoprotein (LDL) cholesterol in serum was measured by TBA-c16000 (Toshiba Medical Systems Corporation, Tochigi, Japan). Lymphatic ducts were obtained from patients with primary breast cancer who underwent sentinel node biopsy or axillary lymph node dissection. Written informed consent was obtained from all patients in accordance with the Declaration of Helsinki. All protocols were approved by the Institutional Review Boards of KKR Sapporo Medical Center Tonan Hospital.

### Antibodies and reagents

The following primary monoclonal antibodies (mAbs) and polyclonal antibodies (pAbs) were used to detect proteins by immunofluorescence labeling and immunoblot analysis and/or to neutralize the effects of leptin and IL-6: mouse anti-leptin mAb (44802), anti-leptin receptor mAb (52263) (R&D Systems, Minneapolis, MN, USA), rat anti-IL-6 mAb (MQ2-13A5; Miltenyi Biotec, Bergisch Gladbach, Germany), mouse anti-phospho STAT3 (Tyr705) mAb (3E2), rabbit anti-STAT3 pAb (D47E7), anti-SOCS3 pAb (Cell Signaling Technology, Danvers, MA, USA), mouse anti-β-actin mAb (E1C605; EnoGene, New York, NY, USA), rabbit anti-claudin-5 mAb (EPR7583), anti-VE-cadherin pAb (Abcam, Cambridge, MA, USA), rabbit anti-occludin pAb, anti-ZO-1 pAb (Invitrogen, Carlsbad, CA, USA), and mouse anti-D2-40 mAb (413451; Nichirei, Tokyo, Japan). Mouse IgG (11711) isotype controls were purchased from R&D Systems. Recombinant human leptin was obtained from R&D Systems. Recombinant human IL-6 was purchased from Peprotech (Rocky Hill, NJ, USA). STAT3 inhibitor was obtained from EMD Millipore (Billerica, MA, USA).

### Cell culture

Human dermal lymphatic endothelial cells (HDLECs) and human umbilical vein endothelial cells (HUVECs) (PromoCell, Heidelberg, Germany) were cultured in Endothelial Cell Basal Medium MV2 containing 5% fetal calf serum (FCS), 5 ng/ml epidermal growth factor (EGF), 10 ng/ml basic fibroblast growth factor (bFGF), 20 ng/ml insulin-like growth factor (IGF), 0.5 ng/ml vascular endothelial growth factor (VEGF) 165, 1 μg/ml ascorbic acid, 0.2 μg/ml hydrocortisone (PromoCell) and 1% Penicillin-Streptomycin-Amphotericin B Suspension (Wako Pure Chemical Industries, Osaka, Japan). HDLECs and HUVECs at passages 3 to 9 were used in this study.

### Ex vivo culture

Human lymphatic ducts were cut into 1-cm pieces, washed with cold PBS, and transferred onto an uncoated 12-well plate (Corning Inc., Corning, NY, USA). The tissues were incubated with leptin or without leptin at 37°C and 5% CO_2_ in 500 μl growth medium supplemented with 1% Penicillin-Streptomycin-Amphotericin B Suspension for four days. The tissues were examined by scanning and transmission electron microscopy.

### Scanning and transmission electron microscopy

For scanning electron microscopy, lymphatic ducts were fixed with 2.5% glutaraldehyde in PBS overnight at 4°C. After several rinses with PBS, they were postfixed in 1% OsO4 at 4°C for 3 h and washed with distilled water followed by being dehydrated through a graded series of ethanol and freeze drying. Samples were subsequently coated with platinum and observed with an S4300 scanning electron microscope (Hitachi, Tokyo, Japan). For transmission electron microscopy, lymphatic ducts were fixed with 2.5% glutaraldehyde and 0.1 M cacodylate buffer, pH 7.3, overnight at 4°C. After washing with 0.1 M cacodylate buffer, pH 7.3, they were postfixed in 1% OsO4 and 1.5% potassium ferrocyanide in 0.1 M cacodylate buffer for 2 h. Samples were subsequently stained with uranyl acetate for 2 h at room temperature, washed, and dehydrated followed by embedding in Epon 812. Ultrathin sections were cut with a diamond knife, stained with lead citrate, and examined with a JEM-1400 transmission electron microscope (JEOL, Tokyo, Japan) at an acceleration voltage of 100 kV.

### Reverse transcription-polymerase chain reaction (RT-PCR)

Total RNA was extracted from cells using TRIzol (Invitrogen) and was reverse-transcribed using oligo(dT) primers (Thermo Fisher Scientific, Marietta, OH, USA). PCR was performed with GoTaq Green Master Mix (Promega, Madison, WI, USA) according to the manufacturer’s instructions, using 25, 30 or 35 cycles with cycle times of 15 s at 96°C, 30 s at 55°C, and 60 s at 72°C. The final elongation time was 7 min at 72°C. Of the 25 μl total PCR products, 10 μl was analyzed in 1% agarose gels following staining with ethidium bromide. To provide a quantitative control for reaction efficiency, PCRs were performed with primers coding for the housekeeping gene glyceraldehyde 3-phosphate dehydrogenase (GAPDH). Primers used to detect Ob-R, IL-6R, and GAPDH are shown in Table 2.

**Table 2:** List of primers for polymerase chain reaction used in this study.

Gene	Forward primer (5’ → 3’)	Reverse primer (5’ → 3’)	Product size (bp)
**Ob-R**	GCCGAAAGCCAGAGACAACC	GCTGGGAATGGGCACGATAC	392
**IL-1**β	GGGCCTCAAGGAAAAGAATC	TTCTGCTTGAGAGGTGCTGA	205
**IL-6**	TACCCCCAGGAGAAGATTCC	TTTTCTGCCAGTGCCTCTTT	175
**IL-8**	GTGCAGTTTTGCCAAGGAGT	CTCTGCACCCAGTTTTCCTT	196
**TNF-α**	CCCCAGGGACCTCTCTCTAA	TGAGGTACAGGCCCTCTGAT	212
**IL-6R**	CATTGCCATTGTTCTGAGGTTC	AGTAGTCTGTATTGCTGATGTC	260
**GAPDH**	GAGTCAACGGATTTGGTCGT	TTGATTTTGGAGGGATCTCG	238

Ob-R, leptin receptor; IL, interleukin; TNF, tumor necrosis factor; IL-6R, IL-6 receptor; GAPDH, glyceraldehyde-3-phosphate dehydrogenase; bp, base pair.

### Quantitative real-time PCR

Quantitative real-time PCR was performed using a TaqMan Gene Expression Assay kit (Life Technologies, Carlsbad, CA, USA) or SYBR Premix Ex Taq (Takara Bio Inc.; Shiga, Japan) with the Roche LightCycler 480 Real-Time PCR Detection System (Roche Diagnostics GmbH, Mannheim, Germany) as described in the protocol of the manufacturer. For the TaqMan-based detection system, the amount of GAPDH mRNA was used to standardize the amount of vascular endothelial growth factor receptor 3 (VEGFR-3; Hs01047677) mRNAs. Sequences of the primer set for SYBR Green-based detection were designed and used in this study (Table 2). The ΔΔCT method was used to calculate the relative mRNA expression of triplicate specimens.

### Enzyme-linked immunosorbent assay (ELISA)

Concentrations of IL-6 in cell-free culture supernatants were analyzed in triplicate with specific ELISA kits (R&D Systems) as described in the manufacturer’s protocol. The detection limit of IL-6 is 10 pg/ml.

### Immunoblotting

For *in vitro* cell cultures, cell lysates were prepared as follows. First, confluent monolayers were collected in RIPA buffer (Wako Pure Chemical Industries) containing protease and phosphatase inhibitor cocktails (Nakarai Tesque, Kyoto, Japan). After sonication, lysates were cleared by centrifugation (5,000 × g at 4°C for 5 min). Protein concentration was determined using a Pierce BCA Protein Assay Kit (Thermo Fisher Scientific). Samples were resuspended with β-ME sample treatment for Tris SDS (Cosmo Bio, Tokyo, Japan) and 15–20 μg of protein was loaded per lane. Immunoblots were quantified using Image J software (National Institutes of Health, Bethesda, MD, USA).

### Immunohistochemistry

Paraffin-embedded specimens were deparaffinized in ethanol and xylene and incubated with 3% H_2_O_2_ for 5 min to quench endogenous peroxidase activity. After microwave treatment (for 20 min with Novocastra Epitope Retrieval Solutions pH 6, Novocastra; Leica Microsystems, Wetzlar, Germany), the sections were incubated in blocking solution (Block Ace; DS Pharma Biomedical, Osaka, Japan) for 30 min before incubation with a primary antibody. Then the sections were incubated with mouse anti-leptin receptor for 15 min, washed, and incubated with a horseradish peroxidase-conjugated anti-mouse secondary antibody (EnVision/HRP; Dako, Glostrup, Denmark) for 8 min. A further washing in PBS was followed by developing in DAB (Dako) as a chromogen for signal visualization. Anti-mouse IgG (Leica, Wetzlar, Germany) was used for a negative control. The slides were counterstained with Mayer's haematoxylin and coverslipped using mounting medium. Consecutive sections of human lymphatic ducts were stained with hematoxylin and eosin (Sakura Finetek Japan, Tokyo, Japan) according to the manufacturer’s instructions. Images were acquired using a TS100 confocal microscope and DS-L2 (Nikon, Tokyo, Japan).

### Tube formation assay

A tube formation assay was performed as previously described [31]. Briefly, HDLECs were harvested after indicated treatments and then seeded into a 24-well plate (2 × 10^5^/well) precoated with 300 μl (9.7 mg/ml) matrigel (Corning) in 300 μl culture medium with or without leptin, IL-6 and/or anti-leptin antibody for neutralization. After 17 h of incubation at 37°C, the capillary tube structures were observed and representative images were captured with an inverted phase-contrast microscope (Olympus, Tokyo, Japan). Total tube length was quantified by NIH Image J software.

### Cell proliferation assay

A WST-8 assay (Dojindo Molecular Technologies, Tokyo, Japan) was performed to analyze HDLEC proliferation as described previously [32]. Briefly, cells were seeded in 96-well plates at a density of 2.5 × 10^3^ cells/well in 100 μl of regular culture media. At 12 h after plating, the media were changed to fresh media with or without leptin, IL-6 anti-leptin, anti-IL-6, and/or their isotype control antibodies for neutralization at 30 min prior to leptin treatment. After 17 h of incubation, 10 μl WST-8 was added to each well and incubated for another 4 h at 37°C in a CO_2_ incubator. Absorbance in each well was measured with a microplate reader (Thermo Fisher Scientific) using a wavelength of 450 nm.

### Statistical analysis

Data are shown as means ± standard deviation (SD) or means ± standard error of the mean (SEM). Significant differences between specimens were determined by using Student’s *t*-test. Probability values less than 0.05 were considered significant.

## Results

### Leptin induces disorganization of human lymphatic ducts

**Fig 1:**
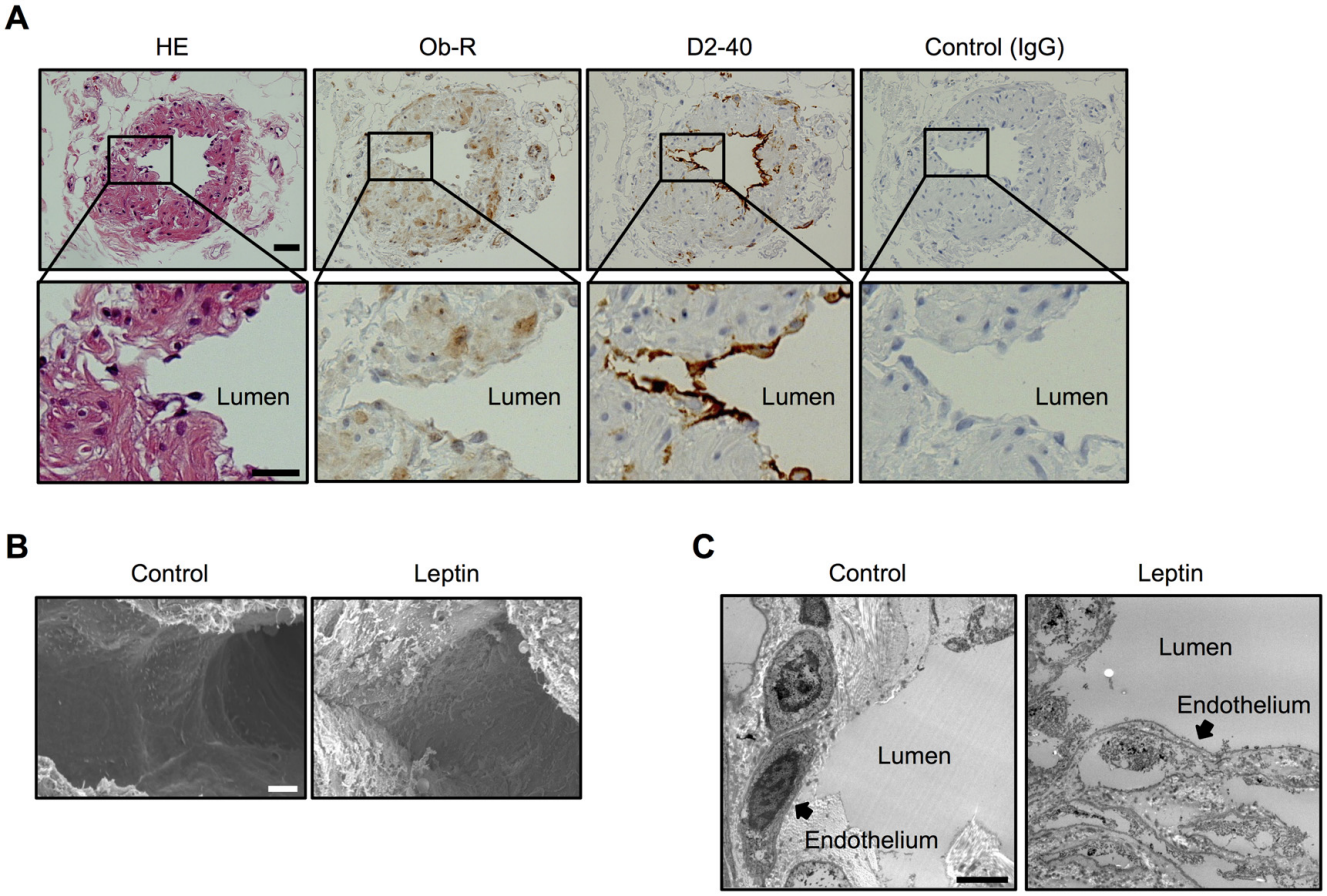
Morphological changes of the human lymphatic duct exposed to leptin. (**A**) Immunohistochemistry showing Ob-R and D2-40 in the human lymphatic duct. Mouse IgG was used as a negative control. Consecutive sections of the human lymphatic duct were stained with hematoxylin and eosin (HE). Scale bar: 5 μm. Scanning electron microscopy (**B**) and transmission electron microscopy (**C**) images demonstrating morphological changes of the human lymphatic duct exposed to a high dose of leptin for three days. Scale bar: 100 μm (**B**), 2 μm (**C**). Images (**B** and **C)** are representative images obtained from at least 4 different lymphatic duct specimens treated with/without leptin.

Previous studies have suggested a relationship between obesity and postoperative lymphedema [10–12]. To confirm the relationship between obesity and postoperative lymphedema, we first characterized patients with breast cancer in our hospital. We divided the patients into two groups, patients with postoperative lymphedema (objective lymphedema) and those without (no lymphedema), and we examined correlations between age, BMI (25 ≤ BMI < 30 and BMI ≥ 30), histological findings (estrogen and progesterone receptor), and serum level of low-density lipoprotein cholesterol. As shown in Table 1, a significant correlation was found between 25 ≤ BMI < 30 or BMI ≥ 30 and existence of lymphedema. However, there were no correlations between estrogen receptor, progesterone receptor, and serum level of LDL cholesterol in the two groups. Since increased serum levels of leptin in obese patients have been reported [18,33], we hypothesized that leptin has an important role in the pathogenesis of postoperative lymphedema in obese patients. To this end, we first studied and confirmed the expression of Ob-R in human lymphatic ducts obtained from patients without lymphedema (Fig 1A). Next, to study the effects of leptin on human lymphatic ducts, they were exposed to leptin and examined by scanning and transmission electron microscopy. Scanning and transmission microscopy images revealed disorganization of lymphatic ducts exposed to leptin compared with the controls (without leptin treatment), suggesting compromised endothelial cells lining lymphatic ducts (Fig 1B and 1C).

### Leptin compromises tube formation and cell proliferation in human lymphatic endothelial cells

**Fig 2:**
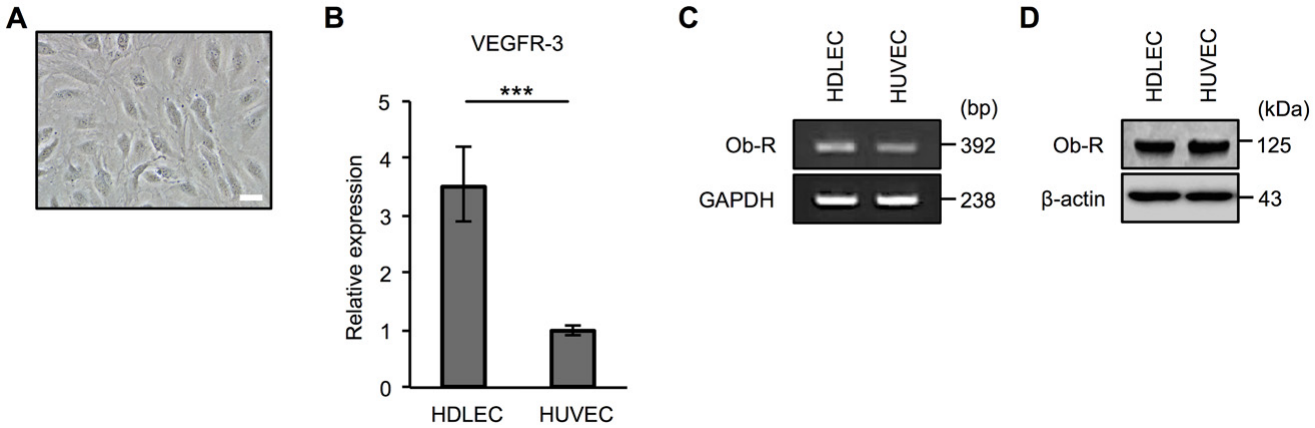
Characteristics of human lymphatic endothelial cells. (**A**) Phase contrast microscopy image showing the morphology of HDLECs used in this study. Scale bar: 1 μm. (**B**) Real-time PCR to confirm the expression of VEGFR-3 mRNA in human HDLECs and HUVECs. Histograms represent the relative expression of VEGFR-3 mRNA. Results are expressed as means ± SEM of three independent experiments; ***p < 0.001, *t*-test. PCR (**C**) and immunoblot analysis (**D**) to confirm the expression of leptin receptor (Ob-R) mRNA (**C**) and protein (**D**) in HDLECs and HUVECs. (**B** and **C**) GAPDH was used as an internal control. bp: base pair. (**D**) β-actin was used as a loading control.

**Fig 3:**
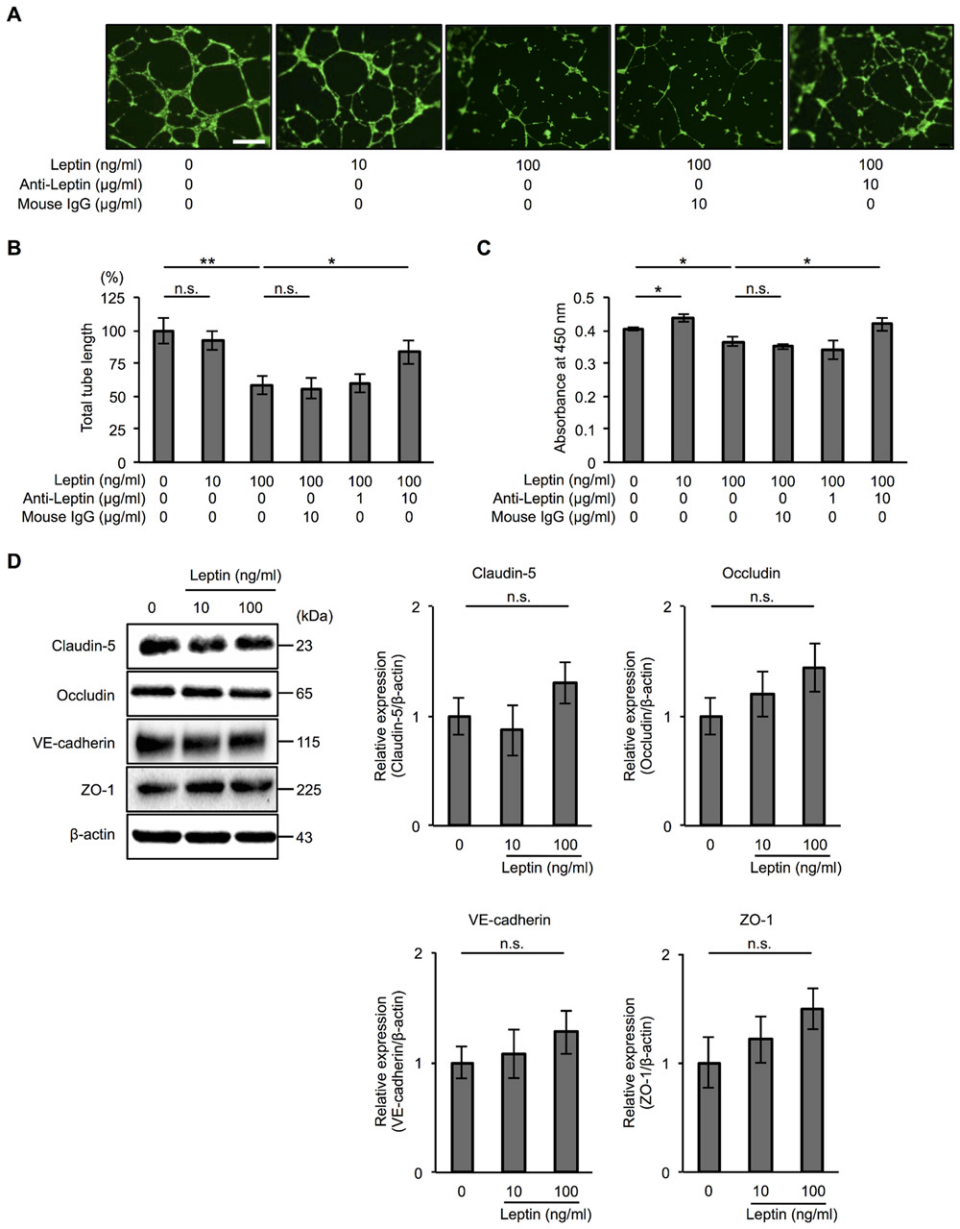
Effect of leptin on lymphatic endothelial cell tube formation and proliferation. (**A**) Representative phase contrast microscopy images showing tube formation in HDLECs treated with leptin (10, 100 ng/ml) for 17 h with or without anti-leptin antibody (10 μg/ml). Scale bar: 50 μm. (**B**) Histograms represent total tube length in HDLECs exposed to leptin (10, 100 ng/ml) for 17 h with or without 1 or 10 μg/ml anti-leptin antibody. Total tube length and mean length in each condition were measured using Image J software. Results are expressed as means ± SEM of four independent experiments; *p < 0.05, **p < 0.01, *t*-test. n.s.: not significant. (**C**) WST-8 assay showing proliferation of HDLECs treated with leptin (10, 100 ng/ml) for 17 h with or without 1 or 10 μg/ml anti-leptin antibody. Histograms represent absorbance at 450 nm. Results are expressed as means ± SEM of four independent experiments; *p < 0.05, *t*-test. n.s.: not significant. (**A-C**) Anti-leptin antibody was added 30 min prior to leptin treatment. Ten μg/ml mouse IgG was used as an isotype control to anti-leptin antibody. (**D**) Immunoblot analysis showing the expression of claudin-5, occludin, VE-cadherin, and ZO-1 in HDLECs treated with leptin (10, 100 ng/ml) for 24 h. β-actin was used as a loading control. Histograms represent relative expression of claudin-5, occludin, VE-cadherin, and ZO-1 proteins in HDLECs exposed to 10 or 100 ng/ml leptin for 24 h as determined by densitometry analysis. Results are expressed as means ± SEM of four independent experiments; *t*-test. n.s.: not significant.

To investigate the mechanism by which leptin compromises the structure of the lymphatic duct, we performed *in vitro* experiments using HDLECs. The morphology of HDLECs is shown in Fig 2A. We first confirmed that the expression level of VEGFR-3 mRNA, which is specifically expressed in lymphatic endothelial cells, was higher in HDLECs than in HUVECs (Fig 2B). A previous study demonstrated that leptin regulates tube formation in vascular endothelial cells [34]. Therefore, we next examined the effect of leptin on tube formation in HDLECs. We confirmed the expression of Ob-R at transcriptional (Fig 2C) and protein levels (Fig 2D) in HDLECs. A tube formation assay revealed that a high dose of leptin (100 ng/ml) inhibited tube formation in HDLECs and that a neutralizing antibody of leptin rescued the effect (Fig 3A and 3B). We also found that a high dose of leptin inhibited cell proliferation and that an anti-leptin antibody neutralized the effect in HDLECs, although 10 ng/ml leptin induced cell proliferation (Fig 3C and S1 Fig). To clarify the mechanism of compromised tube formation by leptin treatment, we analyzed the levels of claudin-5, occludin, VE-cadherin, and ZO-1 proteins, which are constituents of tight and adherence junction complexes and are mainly expressed in lymphatic endothelial cells [35]. Immunoblot analysis revealed that exposure to leptin did not affect these intercellular junctional proteins in HDLECs (Fig 3D). These results indicate that decreased cell proliferation mediated by a high dose of leptin underlies one of the mechanisms by which a high dose of leptin inhibits tube formation in HDLECs without changing the expression of intercellular junctional proteins.

### Effect of leptin on IL-6 expression in human lymphatic endothelial cells

**Fig 4:**
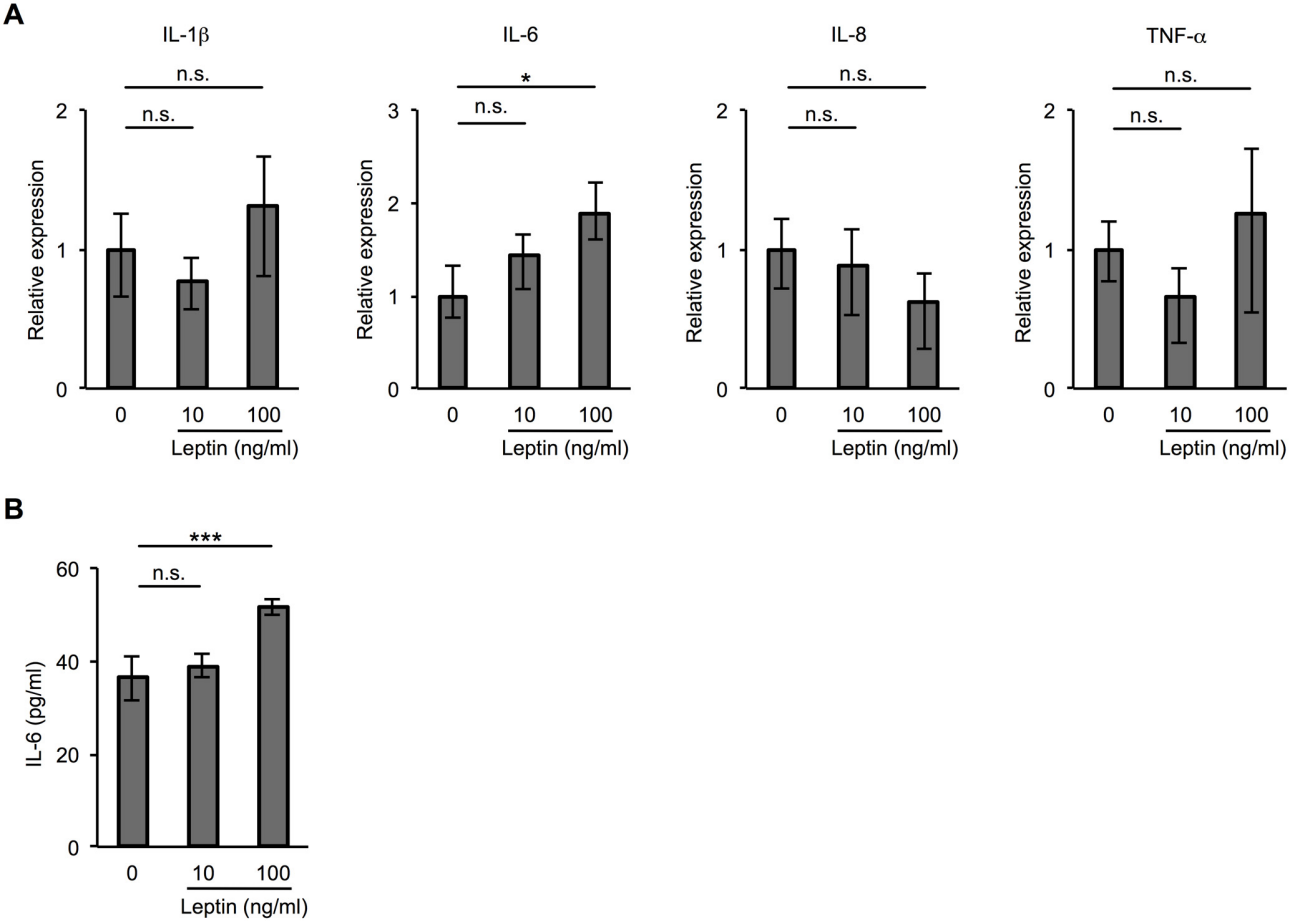
Effect of leptin on IL-6 expression in human lymphatic endothelial cells. (**A**) Real-time PCR showing mRNA expression of proinflammatory cytokines (IL-1β, IL-6, IL-8, and TNF-α) in HDLECs treated with 10 or 100 ng/ml leptin for 2 h. Histograms represent the relative expression of IL-1β, IL-6, IL-8, and TNF-α mRNA. Results are expressed as means ± SEM of three independent experiments; *p < 0.05, *t*-test. n.s.: not significant. GAPDH was used as an internal control. (**B**) ELISA showing the level of IL-6 in human HDLECs treated with 10 or 100 ng/ml leptin for 48 h. Results are expressed as means ± SEM of three independent experiments; ***p < 0.01, *t*-test. n.s.: not significant.

Previous studies have demonstrated that leptin enhances the expression of proinflammatory cytokines such as IL-6 and IL-8 and regulates inflammatory responses [28,29]. To determine whether leptin induces the production of proinflammatory cytokines from HDLECs, we examined the expression of proinflammatory cytokines (IL-1β, IL-6, IL-8, and TNF-α) in HDLECs treated with leptin. As shown in Fig 4A, it was determined by quantitative real-time PCR that exposure to leptin enhanced the expression of IL-6 mRNA, but not that of other proinflammatory cytokines, in HDLECs. We also found by ELISA that 100 ng/ml leptin induced the expression of IL-6 at the protein level (Fig 4B).

### IL-6 has a compensatory effect on leptin-induced modification of tube formation in human lymphatic endothelial cells

**Fig 5:**
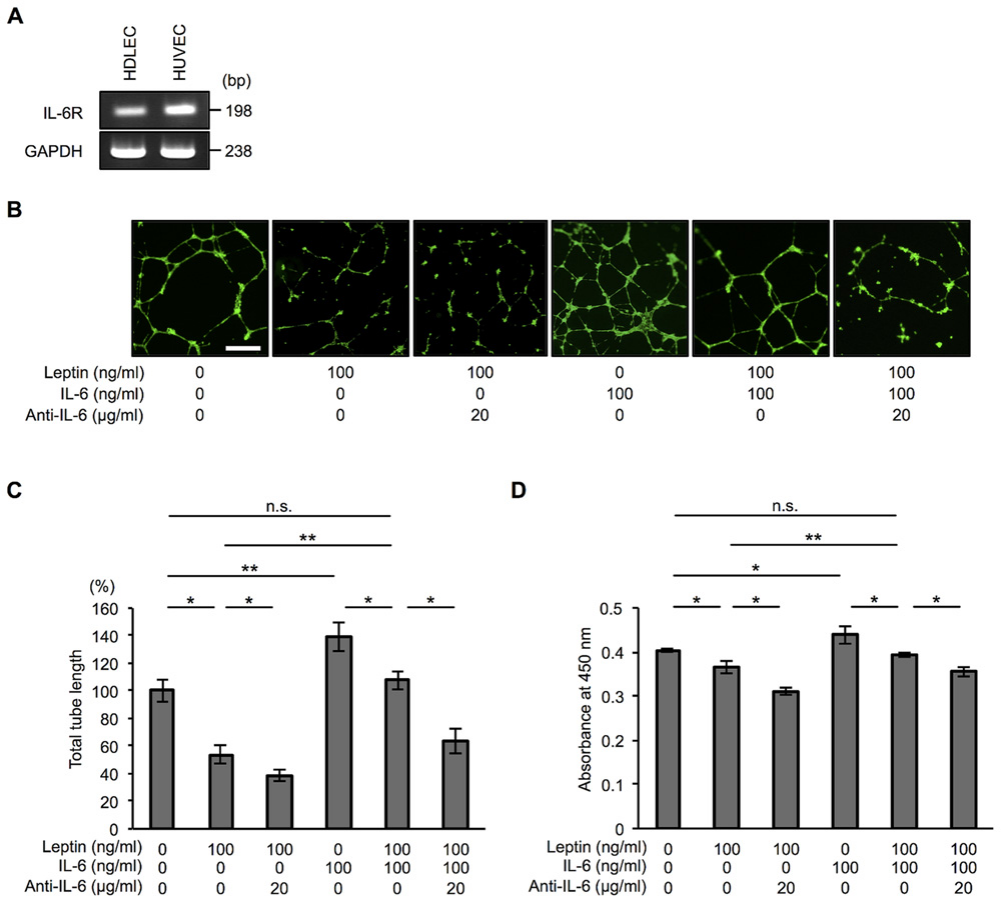
IL-6 has a compensatory effect on leptin-induced modification of tube formation and cell proliferation in human lymphatic endothelial cells. (**A**) PCR to confirm the expression of IL-6 receptor (IL-6R) mRNA in HDLECs and HUVECs. GAPDH was used as an internal control. bp: base pair. (**B**) Representative phase contrast microscopy images showing tube formation in HDLECs treated with 100 ng/ml leptin with or without 20 μg/ml anti-IL-6 antibody, treated with 100 ng/ml IL-6, or treated with a combination of 100 ng/ml leptin and 100 ng/ml IL-6 with or without 20 μg/ml anti-IL-6 antibody for 17 h. Scale bar: 50 μm. (**C**) Histograms represent total tube length in HDLECs exposed to 100 ng/ml leptin with or without anti-IL-6 antibody (20 μg/ml), exposed to 100 ng/ml IL-6, or exposed to a combination of 100 ng/ml leptin and 100 ng/ml IL-6 with or without 20 μg/ml anti-IL-6 antibody for 17 h. Total tube length and mean length in each condition were measured using Image J software. Results are expressed as means ± SEM of four independent experiments; *p < 0.05, **p < 0.01, *t*-test. n.s.: not significant. (**D**) WST-8 assay showing proliferation of HDLECs treated with 100 ng/ml leptin with or without anti-IL-6 antibody (20 μg/ml), treated with 100 ng/ml IL-6, or treated with a combination of 100 ng/ml leptin and 100 ng/ml IL-6 with or without 20 μg/ml anti-IL-6 antibody for 48 h. Histograms represent absorbance at 450 nm. Results are expressed as means ± SEM of four independent experiments; *p < 0.05, **p < 0.01, *t*-test. n.s.: not significant.

Since leptin has been shown to induce expression of the proinflammatory cytokine IL-6, we examined the effect of IL-6 on lymphatic endothelial cell homeostasis. We first confirmed expression of the IL-6 receptor (IL-6R) in HDLECs and HUVECs as a control (Fig 5A). Indeed, treatment with recombinant IL-6 promoted tube formation and proliferation of HDLECs (Fig 5B, 5C and 5D and S1 Fig). Therefore, we hypothesized that IL-6 has a compensatory role in leptin-induced compromise of tube formation in HDLECs. To test our hypothesis, we treated HDLECs with a combination of recombinant IL-6 and high-dose leptin and evaluated their tube formation and cell proliferation. We found that their tube formation and cell proliferation were comparable with those in HDLECs without any treatments, and an anti-IL-6 antibody reduced their tube formation and cell proliferation (Fig 5B, 5C and 5D). We also found that depletion of endogenous IL-6 from leptin-treated HDLECs by treatment with an anti-IL-6 antibody reduced their tube formation and cell proliferation compared with those in leptin-treated cells without anti-IL-6 antibody treatment. Taken together, our results showed that not only did IL-6 rescue leptin-induced compromise of tube formation (Fig 5C) but that it also rescued leptin-induced inhibition of proliferation in HDLECs (Fig 5D), suggesting that IL-6 secreted from HDLECs treated with leptin has a compensatory effect on leptin-induced modification of tube formation in HDLECs.

### Increased SOCS3 underlies the mechanism by which a high dose of leptin inhibits tube formation in human lymphatic endothelial cells

**Fig 6:**
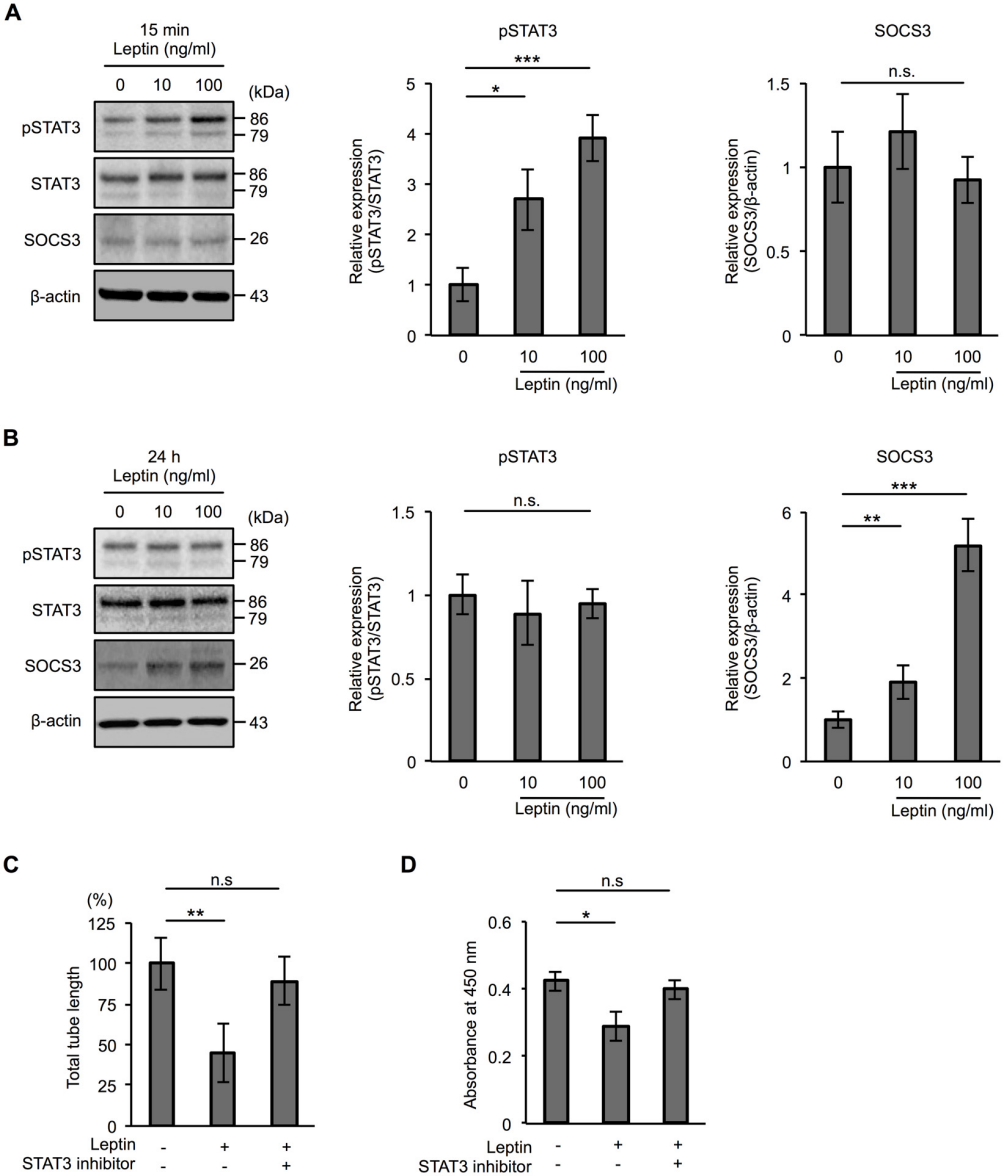
Leptin regulates STAT3 phosphorylation and SOCS3 expression in human lymphatic endothelial cells. Immunoblot analysis of pSTAT3 (Y705), STAT3 and SOCS3 in HDLECs exposed to 10 or 100 ng/ml leptin for 15 min (**A**) and 24 h (**B**). β-actin was used as a loading control. Histograms represent relative expression of pSTAT3 and SOCS3 proteins in HDLECs exposed to 10 or 100 ng/ml leptin for 15 min (**A**) and 24 h (**B**) as determined by densitometry analysis. Results are expressed as means ± SEM of four independent experiments; *p < 0.05, **p < 0.01, ***p < 0.001, *t*-test. n.s.: not significant. (**C**) Histograms represent total tube length in HDLECs exposed to leptin (100 ng/ml) for 17 h with or without 250 nM STAT3 inhibitor. Total tube length and mean length in each condition were measured using Image J software. Results are expressed as means ± SEM of four independent experiments; **p < 0.01, *t*-test. n.s.: not significant. (**D**) WST-8 assay showing proliferation of HDLECs treated with leptin (100 ng/ml) for 17 h with or without 250 nM STAT3 inhibitor. Histograms represent absorbance at 450 nm. Results are expressed as means ± SEM of four independent experiments; *p < 0.05, *t*-test. n.s.: not significant. (**C** and **D**) STAT3 inhibitor was added 30 min prior to leptin treatment.

We next investigated the mechanism by which a high dose of leptin inhibits tube formation. Leptin regulates cellular homeostasis by activating multiple intracellular signaling pathways such as JAK/STAT, MAPK, and PI3K via Ob-Rs [21–23]. Since the JAK/STAT signaling pathway is thought to be main signaling pathway of leptin, we focused on JAK/STAT signaling pathway activation. As shown in Fig 6A, immunoblot analysis demonstrated that leptin increased the phosphorylation of STAT3 (Tyr705) in a dose-dependent manner at 15 min after treatment (Fig 6A). Cumulative evidence has shown that the JAK/STAT pathway of cytokine signaling is under the negative-feedback control of SOCS proteins [36,37]. In addition, leptin has been reported to induce SOCS3 expression [38]. We therefore examined whether leptin increases SOCS3 in HDLECs. We did not see any difference in the level of SOCS3 at 15 min after treatment with leptin (Fig 6A). However, we found an increased level of SOCS3 in HDLECs exposed to leptin for 24 h when STAT3 phosphorylation was not increased (Fig 6B). To determine whether the STAT3 signaling pathway is responsible for the functions of leptin-treated HDLECs, we performed a tube formation assay and a cell proliferation assay using a STAT3-specific inhibitor. As shown in Fig 6C and 6D, the STAT3 inhibitor rescued the effect of leptin on tube formation and cell proliferation in HDLECs. These findings indicate that leptin increases SOCS3 via phosphorylation of STAT3 at an early time point, which is one of the mechanisms by which a high dose of leptin inhibits cell proliferation and/or tube formation of HDLECs.

## Discussion

Leptin regulates various physiological processes and behaviors including appetite, body weight, neuroendocrine functions and glycemia [15,16]. Increased serum levels of leptin in obese patients have been reported [39–42]. Based on those reports, we used leptin at 100 ng/ml as a representative concentration seen in obese patients and at 10 ng/ml in healthy subjects in this study. Our results suggest that not only ‘obese’ patients (BMI ≥ 30) but also ‘overweight’ patients (25 ≤ BMI ≤ 30) are prone to have postoperative lymphedema (Table 1), being consistent with previous reports [13,14]. Leptin is also known to regulate cellular homeostasis such as tube formation and cell proliferation. For example, previous studies demonstrated that leptin induces tube formation in vascular endothelial cells [34] and first-trimester extravillous trophoblast cells [43]. It was also shown that leptin increased proliferation and reduced apoptosis of HUVECs [25,26]. However, the role of leptin in lymphatic endothelial cell homeostasis is not well understood. Therefore, we hypothesized that the mechanism by which postoperative lymphedema is likely to develop in obese patients underlies hyperleptinemia and that it compromises lymphatic endothelial cell homeostasis such as tube formation and cell proliferation. Here we reported that a large amount of leptin (100 ng/ml) inhibited tube formation and cell proliferation in HDLECs. Interestingly, 10 ng/ml leptin did not affect tube formation and increased cell proliferation in HDLECs (Fig 3). These findings are consistent with the results of previous studies in which a physiological concentration of leptin (< 100 ng/ml) increased cell proliferation and the effect was observed with up to 50 and 100 ng/ml leptin in primary stromal vascular cells [44] and in a breast cancer cell line [45], respectively. These findings indicate that an excessive amount of leptin compromises lymphatic endothelial cell homeostasis, while a physiological concentration of leptin is required for maintenance of cellular homeostasis.

Previous studies showed that leptin-induced local inflammation in the vascular endothelium is involved in the development of advanced atherosclerotic lesions [46,47]. It was also shown that leptin enhances the expression of proinflammatory cytokines and regulates inflammatory responses [28,29]. Therefore, we hypothesized that proinflammatory cytokines induced by leptin mediate lymphatic endothelial cell homeostasis. Our results showing that leptin induced IL-6 expression in HDLECs (Fig 4) are consistent with results of previous studies using other cells [28,48]. In this study, we also found that IL-6 rescues the tube formation and cell proliferation worsened by leptin treatment in HDLECs. IL-6 is known as a proinflammatory cytokine that induces production of chemokines [49,50] and regulates immune cells [51]. Our finding suggests that IL-6 has biphasic effects of not only accelerating inflammatory responses but also protecting cellular functions from damage caused by inflammation.

**Fig 7:**
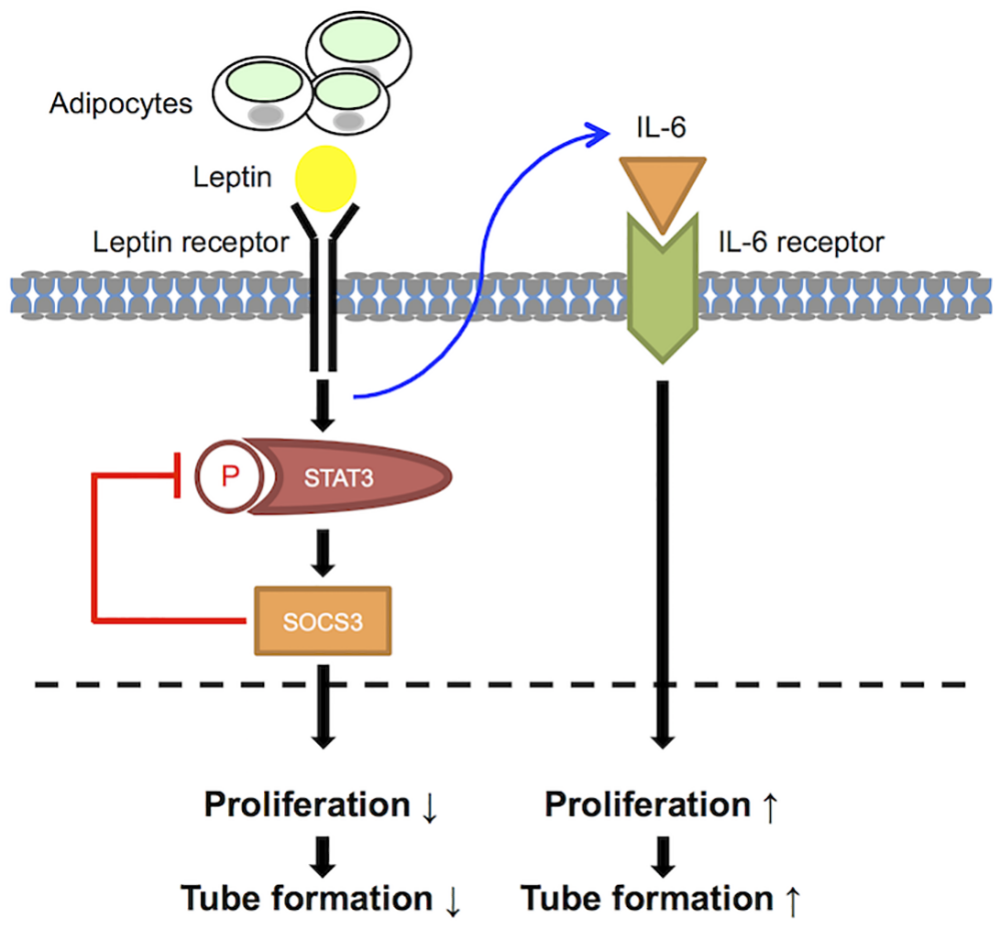
A model for how leptin and IL-6 regulate tube formation and cell proliferation in human lymphatic endothelial cells. Leptin derived from adipocytes regulates tube formation through controlling proliferation of HDLECs. Although leptin activates the STAT3 signaling pathway, which is downstream signaling of Ob-R, at an early time point, STAT3 phosphorylation is not increased at a late time point when SOCS3 proteins are increased by leptin. As a result, proliferation of HDLECs is decreased, leading to inhibition of tube formation. Additionally, SOCS3 induced by leptin is under the regulation of STAT3. Leptin also enhanced expression of the proinflammatory cytokine IL-6 in HDLECs. IL-6 rescues the compromised proliferation and tube formation caused by leptin in an autocrine or paracrine manner.

Leptin regulates cellular function via activation of multiple intracellular signaling pathways including JAK/STAT and MAPK [21–23]. As was observed in previous studies, we observed phosphorylation of STAT3 in HDLECs treated with leptin for 15 min in a dose-dependent manner (Fig 6). We also found opposite effects of leptin on cell proliferation and tube formation in HDLECs depending on the concentration of leptin (Fig 3). However, the mechanism by which a high dose of leptin inhibits cell proliferation and tube formation in HDLECs remains unknown. One of the proposed mechanisms is a change of junctional proteins in HDLECs caused by leptin treatment. The tight junction protein claudin-5 and the adherence junction protein VE-cadherin are known to regulate not only barrier properties but also angiogenesis in vascular endothelial cells [52]. In addition, leptin decreases the levels of tight junction proteins such as zonula occludens-3, claudin-5 and occludin via a leptin receptor-dependent signaling pathway in intestinal epithelial cells [53]. Therefore, we examined the levels of claudin-5, occludin, VE-cadherin, and ZO-1 proteins in HDLECs exposed to leptin. However, we could not see any differences in these intercellular junctional proteins in leptin-treated HDLECs (Fig 3D). Another proposed mechanism links decreased cell proliferation and tube formation mediated by a high dose of leptin in HDLECs to changes of signaling molecules. Previous studies have shown that the JAK/STAT signaling pathway is negatively regulated by SOCS proteins [36,37] and that leptin induces SOCS3 expression [38]. Therefore, we hypothesized that a high dose of leptin induces the expression of SOCS3 proteins, leading to inhibition of the STAT3 signaling pathway. We found that pSTAT3 phosphorylation did not increase in HDLECs at 24 h after treatment with a high dose of leptin when SOCS3 proteins increased in HDLECs exposed to 10 and 100 ng/ml leptin (Fig 6B). We further examined the regulation of SOCS3 induced by leptin in our system and found that the STAT3 inhibitor decreased the level of SOCS3 protein, suggesting that SOCS3 is regulated by the JAK/STAT3 pathway (Fig 7 and S2 Fig). Additionally, we observed that treatment with IL-6 induces phosphorylation of STAT3 in HDLECs (data not shown) as seen in HDLECs treated with leptin (Fig 6A). IL-6 is also known to regulate the JAK/STAT pathway and induce SOCS3 proteins in other cell lines such as human microvascular endothelial cells and T lymphocytes [54,55]. However, unlike the response of leptin to SOCS3 protein, IL-6 did not increase SOCS3 protein in HDLECs even though it induced phosphorylation of STAT3 in our system (data not shown). From these results, Ob-Rs and IL-6Rs have different mechanisms in their downstream signaling by which leptin and IL-6 regulate SOCS3 protein. Further study is needed to clarify these mechanisms. Taken together, the results indicate that leptin increases SOCS3 via phosphorylation of STAT3 at an early time point, which is one of the mechanisms by which a high dose of leptin inhibits cell proliferation and/or tube formation of HDLECs.

In conclusion, our study provides novel insights into the compensatory mechanism by which the proinflammatory cytokine IL-6 rescues the compromised lymphatic endothelial cell homeostasis caused by a superphysiological concentration of leptin in an autocrine or a paracrine manner. In addition to weight control to reduce the serum level of leptin and leptin resistance, regulating the level of IL-6 may also be a viable strategy to treat postoperative lymphedema.

## Supporting Information

S1 Fig Cell counting showing proliferation of HDLECs treated with 100 ng/ml leptin or 100 ng/ml IL-6. Briefly, 1.5 × 10^5^ HDLECs were plated and cultured on a 60-mm cell culture dish for 24 h in the same condition as that used in this study. After 24-h culture, the cells were treated with 100 ng/ml leptin or 100 ng/ml IL-6 for 17 h. They were trypsinized and detached from the culture dish. They were counted under the microscope and the absolute cell number was calculated. Histograms represent cell number (×10^5^). Results are expressed as means ± SEM of four independent experiments; *p < 0.05, *t*-test.
(TIFF)

S2 Fig Immunoblot analysis of SOCS3 in HDLECs exposed to leptin (100 ng/ml) for 24 h with or without 250 nM STAT3 inhibitor. β-actin was used as a loading control. Histograms represent relative expression of SOCS3 proteins in HDLECs exposed to leptin (100 ng/ml) for 24 h with or without 250 nM STAT3 inhibitor as determined by densitometry analysis. Results are expressed as means ± SEM of four independent experiments; *p < 0.05, *t*-test.
(TIFF)
