# Supplementary figures and images for: Novel Mechanisms of Compromised Lymphatic Endothelial Cell Homeostasis in Obesity: The Role of Leptin in Lymphatic Endothelial Cell Tube Formation and Proliferation
Source: PLoS One. 2016 Jul 1;11(7):e0158408. doi: 10.1371/journal.pone.0158408 (PMC4930203; doi:10.1371/journal.pone.0158408)

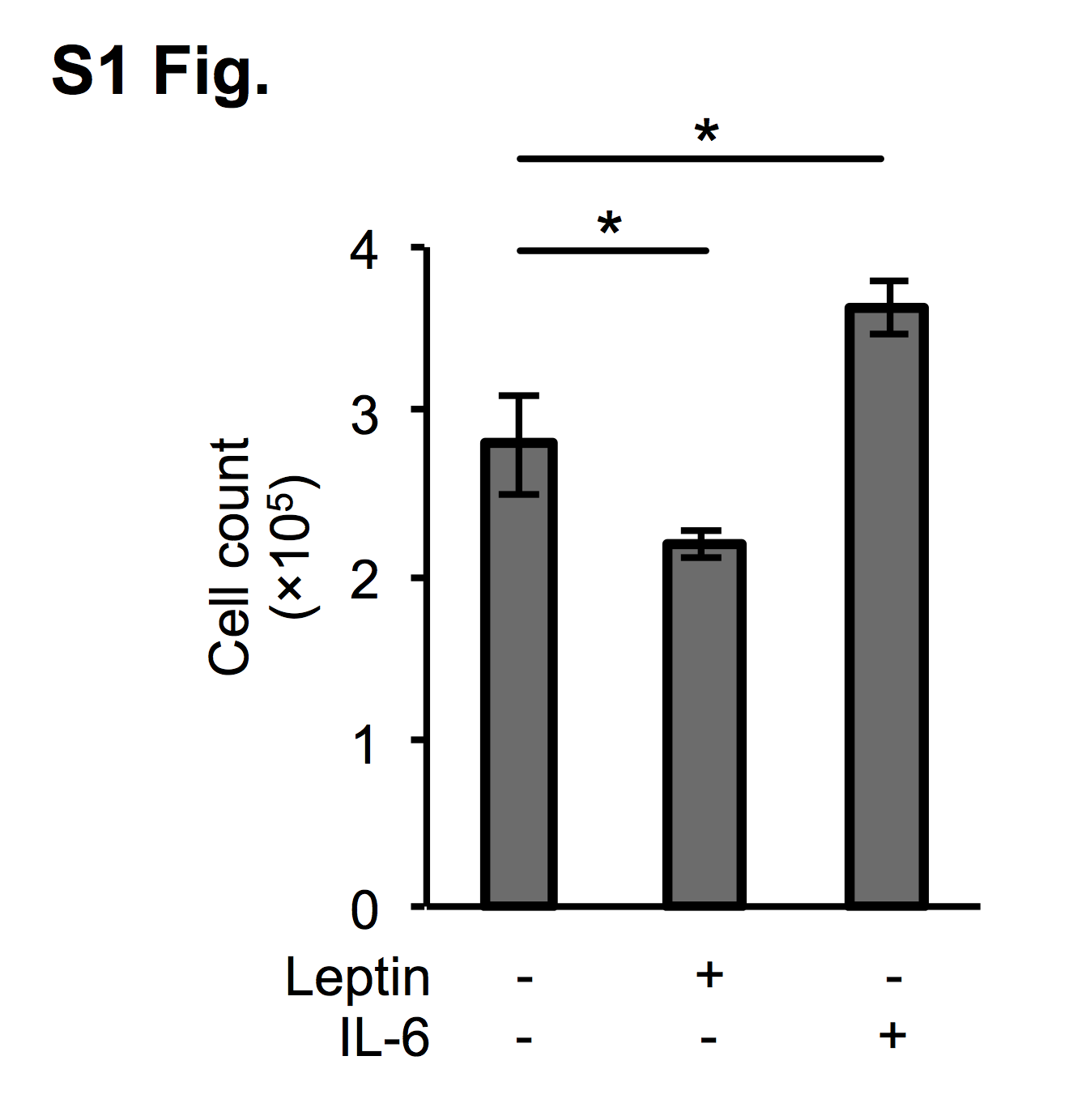

Supplement: S1 Fig — Briefly, 1.5 × 10^5^ HDLECs were plated and cultured on a 60-mm cell culture dish for 24 h in the same condition as that used in this study. After 24-h culture, the cells were treated with 100 ng/ml leptin or 100 ng/ml IL-6 for 17 h. They were trypsinized and detached from the culture dish. They were counted under the microscope and the absolute cell number was calculated. Histograms represent cell number (×105). Results are expressed as means ± SEM of four independent experiments; *p < 0.05, t-test. (TIFF) [file pone.0158408.s001.tiff]

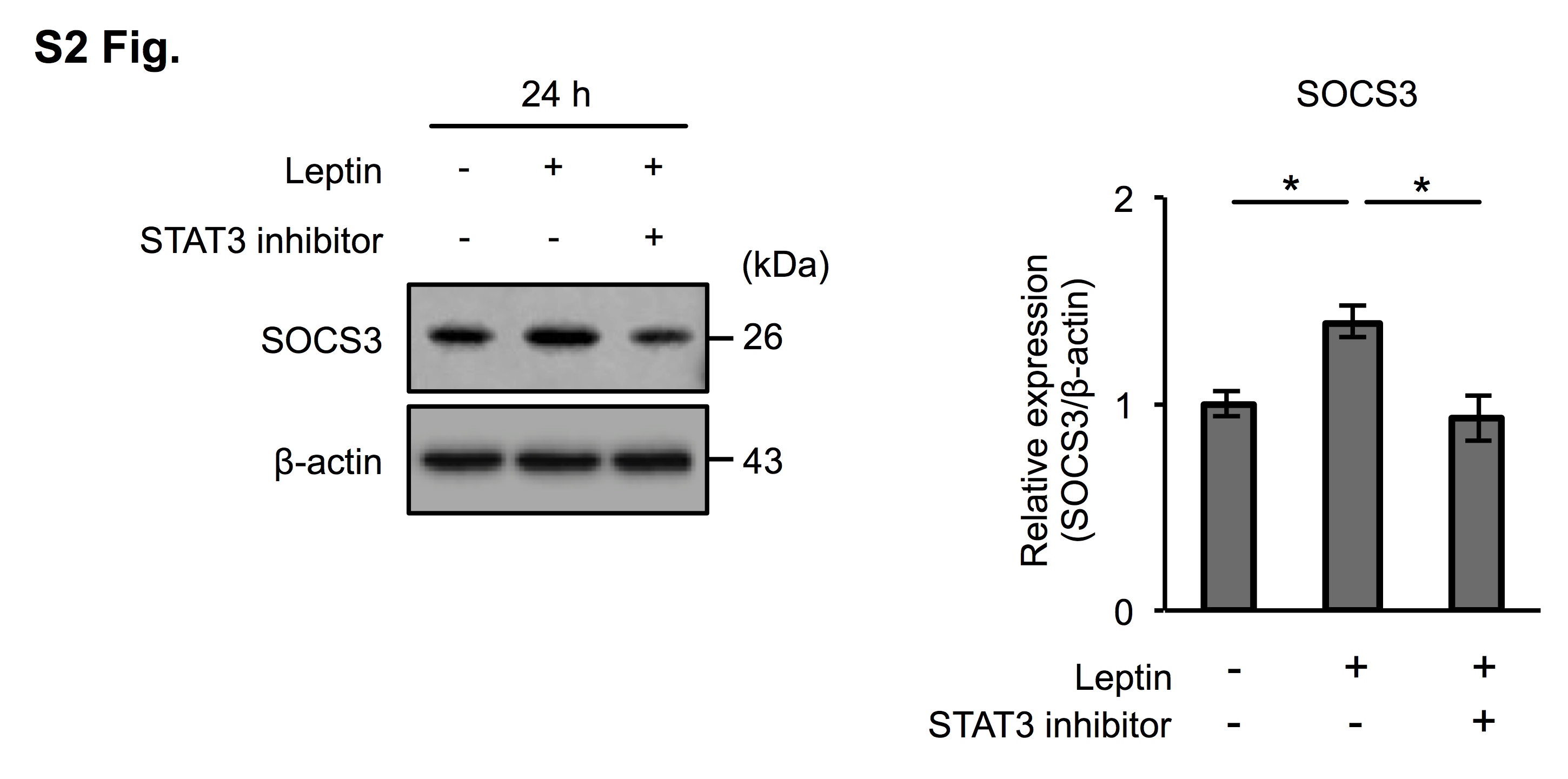

Supplement: S2 Fig — β-actin was used as a loading control. Histograms represent relative expression of SOCS3 proteins in HDLECs exposed to leptin (100 ng/ml) for 24 h with or without 250 nM STAT3 inhibitor as determined by densitometry analysis. Results are expressed as means ± SEM of four independent experiments; *p < 0.05, t-test. (TIFF) [file pone.0158408.s002.tiff]
